# Mannitol versus hypertonic saline for brain relaxation in patients undergoing craniotomy

**DOI:** 10.1590/1516-3180.20151332T2

**Published:** 2014-11-28

**Authors:** Hemanshu Prabhakar, Gyaninder Pal Singh, Vidhu Anand, Mani Kalaivani

## Abstract

**BACKGROUND:**

Patients with brain tumour usually suffer from increased pressure in the skull due to swelling of brain tissue. A swollen brain renders surgical removal of the brain tumour difficult. To ease surgical tumour removal, measures are taken to reduce brain swelling, often referred to as brain relaxation. Brain relaxation can be achieved with intravenous fluids such as mannitol or hypertonic saline. This review was conducted to find out which of the two fluids may have a greater impact on brain relaxation.

Objectives: To compare the effects of mannitol versus those of hypertonic saline on intraoperative brain relaxation in patients undergoing craniotomy.

**METHODS:**

*Search methods:* We searched the Cochrane Central Register of Controlled Trials (CENTRAL) (2013, Issue 10), Medline via Ovid SP (1966 to October 2013) and Embase via Ovid SP (1980 to October 2013). We also searched specific websites, such as www.indmed.nic.in, www.cochrane-sadcct.org and www.Clinicaltrials.gov.

*Selection criteria:* We included randomized controlled trials (RCTs) that compared the use of hypertonic saline versus mannitol for brain relaxation. We also included studies in which any other method used for intraoperative brain relaxation was compared with mannitol or hypertonic saline. Primary outcomes were longest follow-up mortality, Glasgow Outcome Scale score at three months and any adverse events related to mannitol or hypertonic saline. Secondary outcomes were intraoperative brain relaxation, intensive care unit (ICU) stay, hospital stay and quality of life.

*Data collection and analysis:* We used standardized methods for conducting a systematic review, as described by the Cochrane Handbook for Systematic Reviews of Interventions. Two review authors independently extracted details of trial methodology and outcome data from reports of all trials considered eligible for inclusion. All analyses were made on an intention-to-treat basis. We used a fixed-effect model when no evidence was found of significant heterogeneity between studies, and a random-effects model when heterogeneity was likely.

**MAIN RESULTS:**

We included six RCTs with 527 participants. Only one RCT was judged to be at low risk of bias. The remaining five RCTs were at unclear or high risk of bias. No trial mentioned the primary outcomes of longest follow-up mortality, Glasgow Outcome Scale score at three months or any adverse events related to mannitol or hypertonic saline. Three trials mentioned the secondary outcomes of intraoperative brain relaxation, hospital stay and ICU stay; quality of life was not reported in any of the trials. Brain relaxation was inadequate in 42 of 197 participants in the hypertonic saline group and in 68 of 190 participants in the mannitol group. The risk ratio for brain bulge or tense brain in the hypertonic saline group was 0.60 (95% confidence interval (CI) 0.44 to 0.83, low-quality evidence). One trial reported ICU and hospital stay. The mean (standard deviation (SD)) duration of ICU stay in the mannitol and hypertonic saline groups was 1.28 (0.5) and 1.25 (0.5) days (P value 0.64), respectively; the mean (SD) duration of hospital stay in the mannitol and hypertonic saline groups was 5.7 (0.7) and 5.7 (0.8) days (P value 1.00), respectively.

**AUTHORS’ CONCLUSIONS:**

From the limited data available on the use of mannitol and hypertonic saline for brain relaxation during craniotomy, it is suggested that hypertonic saline significantly reduces the risk of tense brain during craniotomy. A single trial suggests that ICU stay and hospital stay are comparable with the use of mannitol or hypertonic saline. However, focus on other related important issues such as long-term mortality, long-term outcome, adverse events and quality of life is needed.

This is the abstract of a Cochrane Review published in the Cochrane Database of Systematic Reviews (CDSR) 2014, issue 7. Art. No.: CD010026. DOI: 10.1002/14651858.CD010026.pub2. For full citation and authors’ details see reference 1.

The abstract of this review is available from: http://onlinelibrary.wiley.com/doi/10.1002/14651858.CD010026.pub2/abstract.
